# Repair and Reinforcement of Implant-Supported Hybrid Denture Using the Running Inlay Technique

**DOI:** 10.7759/cureus.88653

**Published:** 2025-07-24

**Authors:** N Ravi Kumar, Ananthesh H S, Pavithra K Ramanna, Megna Raju

**Affiliations:** 1 Prosthodontics and Crown and Bridge, The Oxford Dental College, Bengaluru, IND

**Keywords:** acrylic, hybrid denture, implant-supported prosthesis, metal inlay, occlusal index

## Abstract

Edentulism is a prevalent issue among elderly individuals. The aim of giving treatment to elderly patients is to provide them with utmost comfort, function, and aesthetics. An implant-supported discovery hybrid prosthesis is an effective way to achieve this. However, a common issue with hybrid prostheses is the fracturing of the acrylic portion. This case report elaborates on the management of a 53-year-old female patient who presented to our department with a fractured hybrid denture. It also highlights a simple, cost-effective way to repair the fractured denture using an occlusal index as a guide and a running metal inlay to hold the prosthesis together, which was secured with the help of cold-cure resin.

## Introduction

Chronic edentulism can impair a person's quality of life. For instance, a patient's inadequate chewing habit limits their food options and jeopardizes their nutrient intake. Moreover, its impact on speech and appearance may make it more difficult to interact with others [1].

The primary goal of implant therapy is to either increase the stability and retention of detachable complete dentures or allow implant-supported fixed prostheses. These are essentially two methods for an implant-supported fixed prosthesis [2].

The hybrid prosthesis is a term commonly used to describe a prosthesis in which acrylic resin and denture teeth are typically supported by a titanium alloy or noble metal bar [1].

Because of its acrylic base and denture teeth, the hybrid denture has several advantages, such as lowering the effect of dynamic occlusal force, being inexpensive, having a high aesthetic appeal, reproducing gingival color, being easier to repair in the event of a porcelain fracture because replacing the denture tooth carries less risk than adding porcelain to a traditional porcelain-metal restoration, and being able to be used with a combination of tilted and axially placed implants, among other abutments due to bone loss.

Despite their 10-15-year success rates, hybrid dentures require regular maintenance and repair to remain stable and functional in the mouth. This is because they can develop several problems. This increased frequency of tooth fractures is caused by the inadequate mechanical retention for teeth and acrylic in the metal, bar/framework, the opposing dentition, the length and presence of the cantilever, the type of occlusion, the material used in the opposing prosthesis, the fewer implants (because fewer implants increase the stress concentration in the abutment/bar) [3].

The literature available on the repair of fractured acrylic portions of hybrid dentures is limited. This clinical report presents an effective method for repairing a fractured acrylic portion of a hybrid denture.

## Case presentation

This clinical report is of a 53-year-old female patient who presented to the department with a fractured lower denture. On further evaluation, the patient mentioned that they had a denture made 10 months prior. On examination, the patient had an implant-supported hybrid prosthesis with respect to the mandible and implant implant-supported over-denture with respect to the maxillary arch.

**Figure 1:**
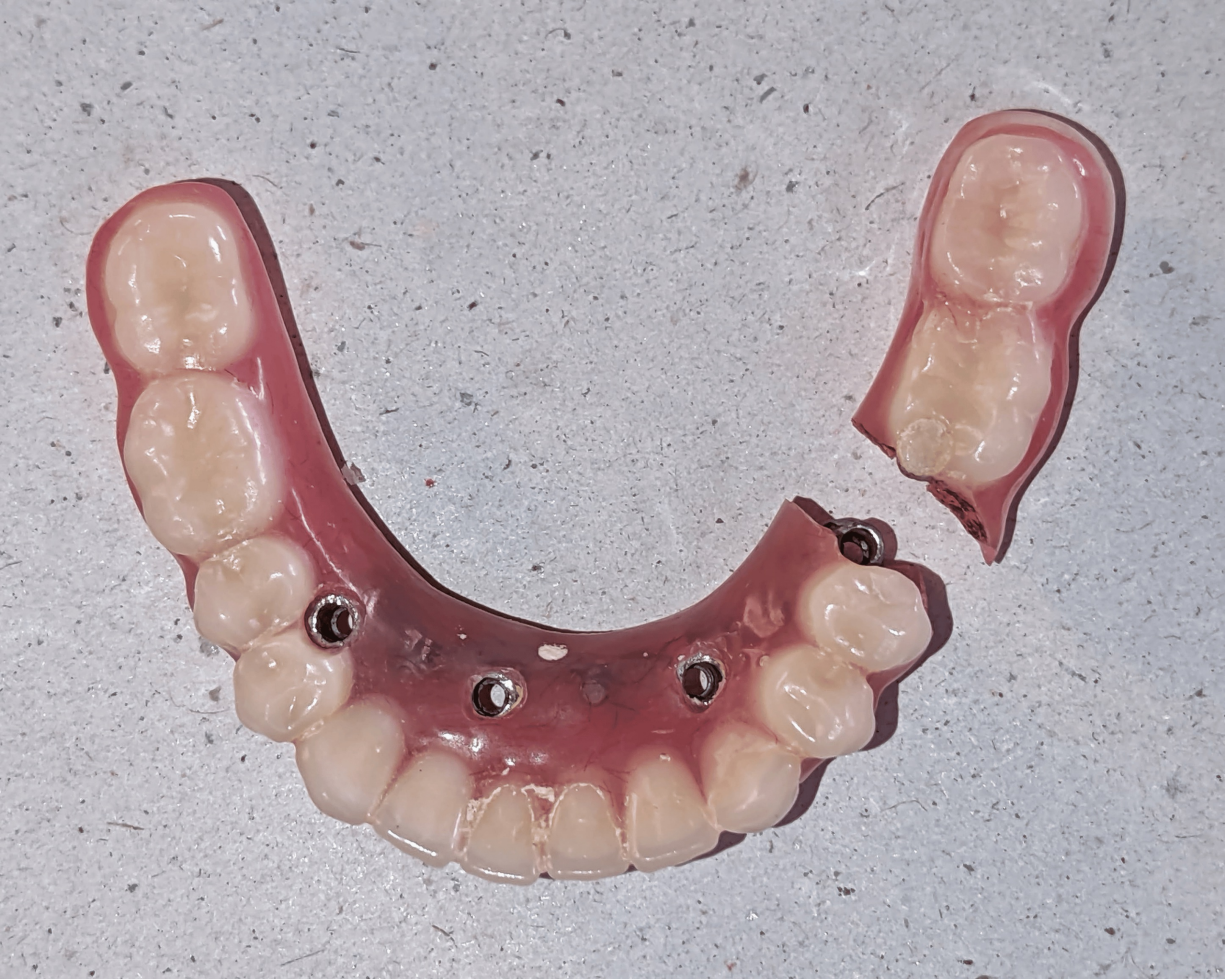
Fractured acrylic portion of the mandibular hybrid denture in between the premolar (20) and molar (19) regions.

**Figure 2:**
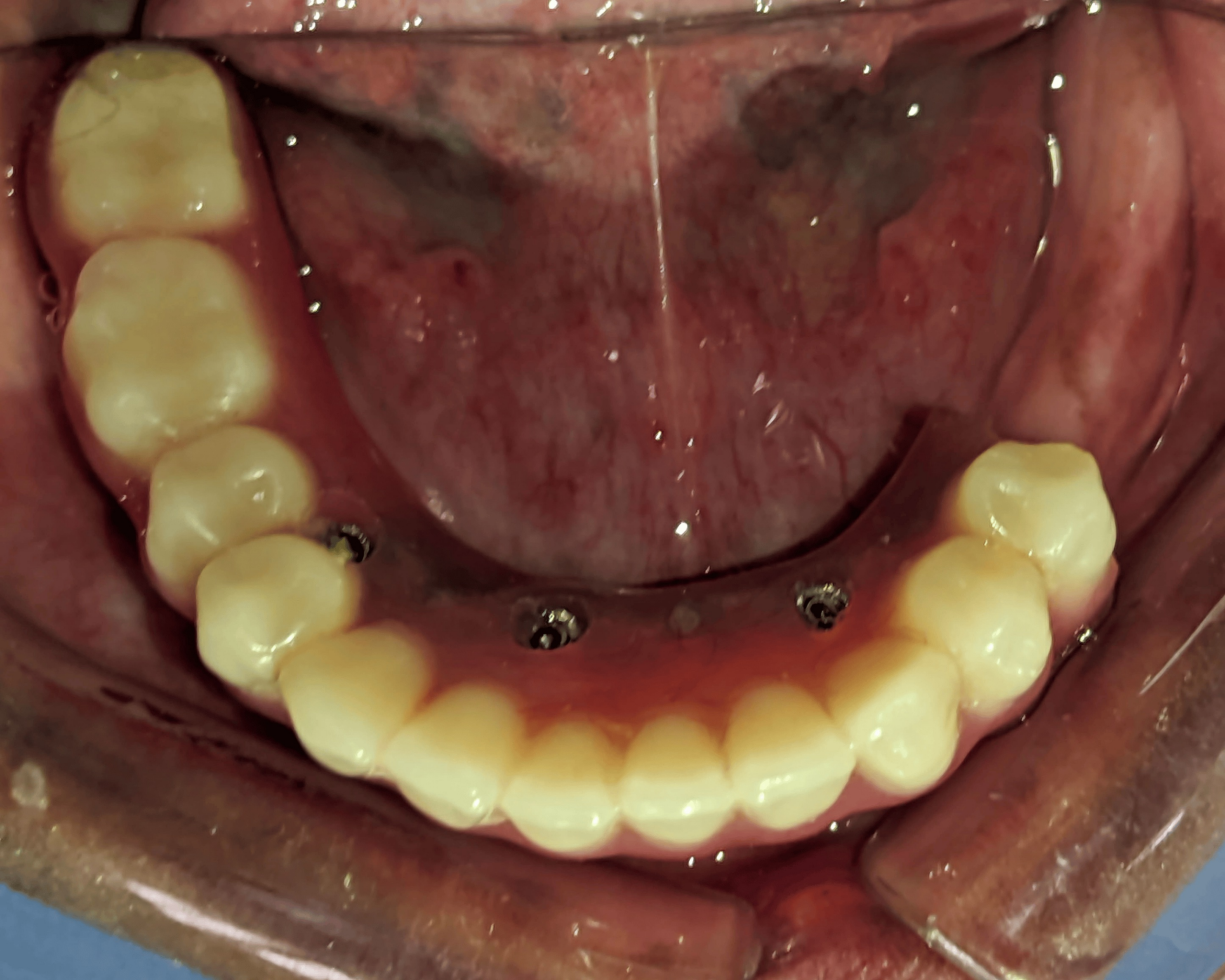
Fractured acrylic portion of the hybrid denture in between the premolar and molar regions (intraoral view).

On further examination, the framework was intact, and the fracture was seen in the acrylic portion of the left side of the mandibular denture in the region between the premolar (20) and molar (19) regions (Figures 1-2).

**Figure 3:**
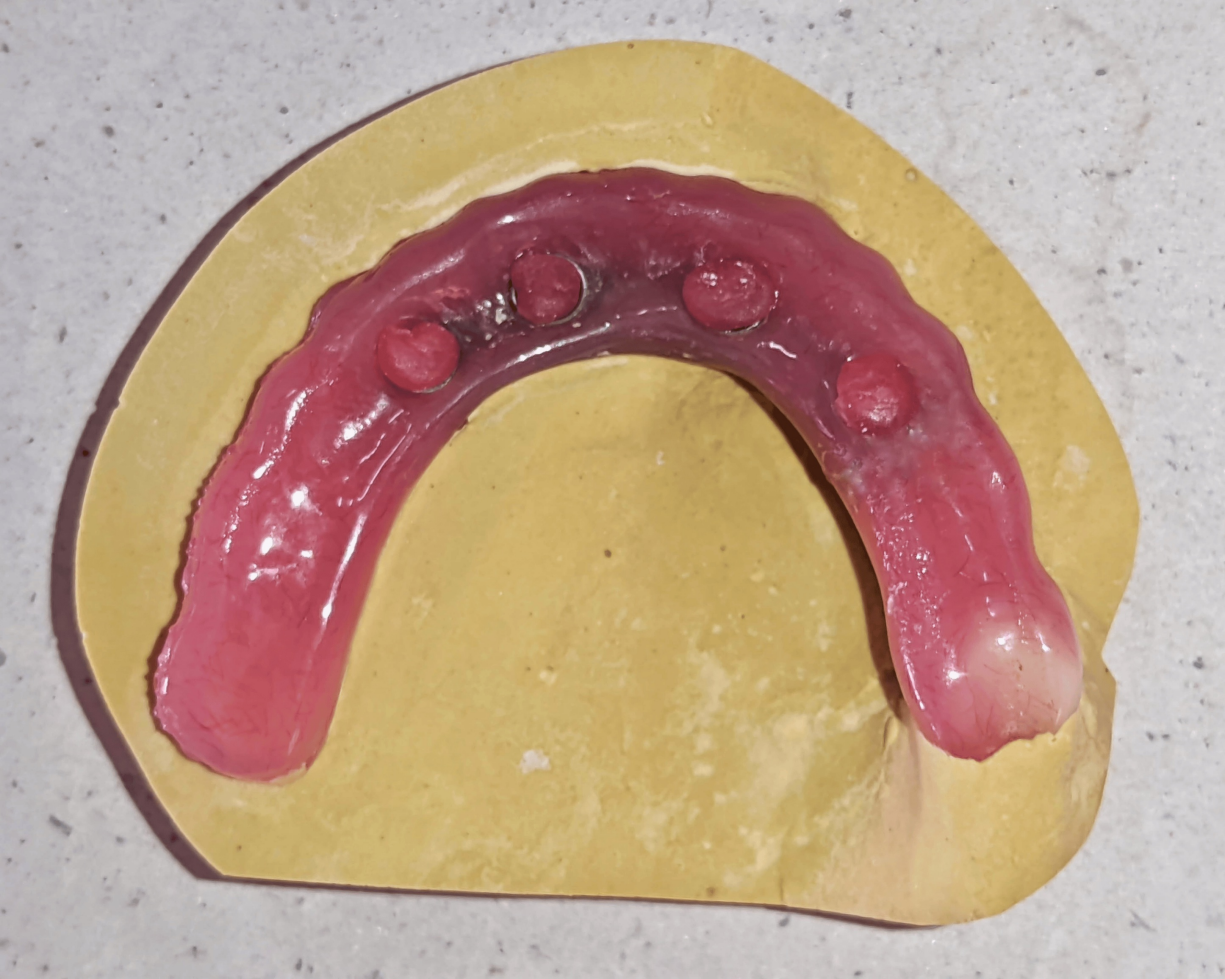
An occlusal index is made with type III dental stone.

On further examination and followed by a discussion with the patient regarding the condition, it was decided to correct the existing prosthesis. Hybrid prosthesis was removed, and healing abutments were placed. The fractured portion of the denture was stabilized with the help of sticky wax. An occlusal index (Figure 3) was made with the help of type A III dental stone (gold stone type III).

**Figure 4:**
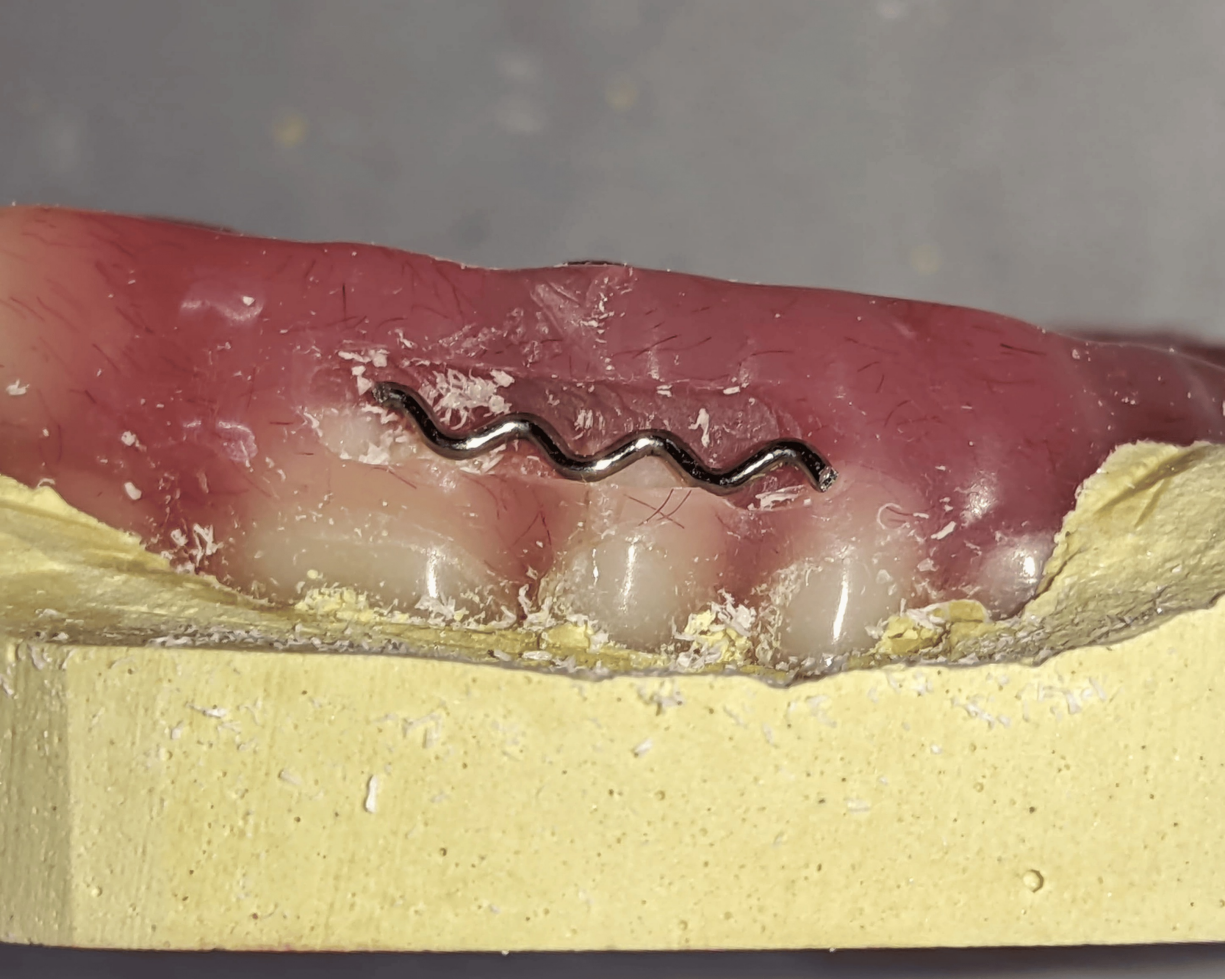
An 0.8 mm orthodontic that was shaped into a zigzag pattern and was placed in the groove and was secured with the help of cold-cure acrylic resin.

The fracture lines were widened, and horizontal grooves were placed along the fractured portion. An 0.8 mm orthodontic wire (Dentaflex stainless steel orthodontic wire, 15 g - 21 SWG/0.8 mm; Dentaurum, Ispringen, Germany) that was shaped into a zigzag pattern (Figure 4) was placed in the groove and secured with the help of cold-cure acrylic resin (Dental DPI RR Cold Cure; Bombay Burmah Trading Corporation Ltd., Mumbai, India).

**Figure 5:**
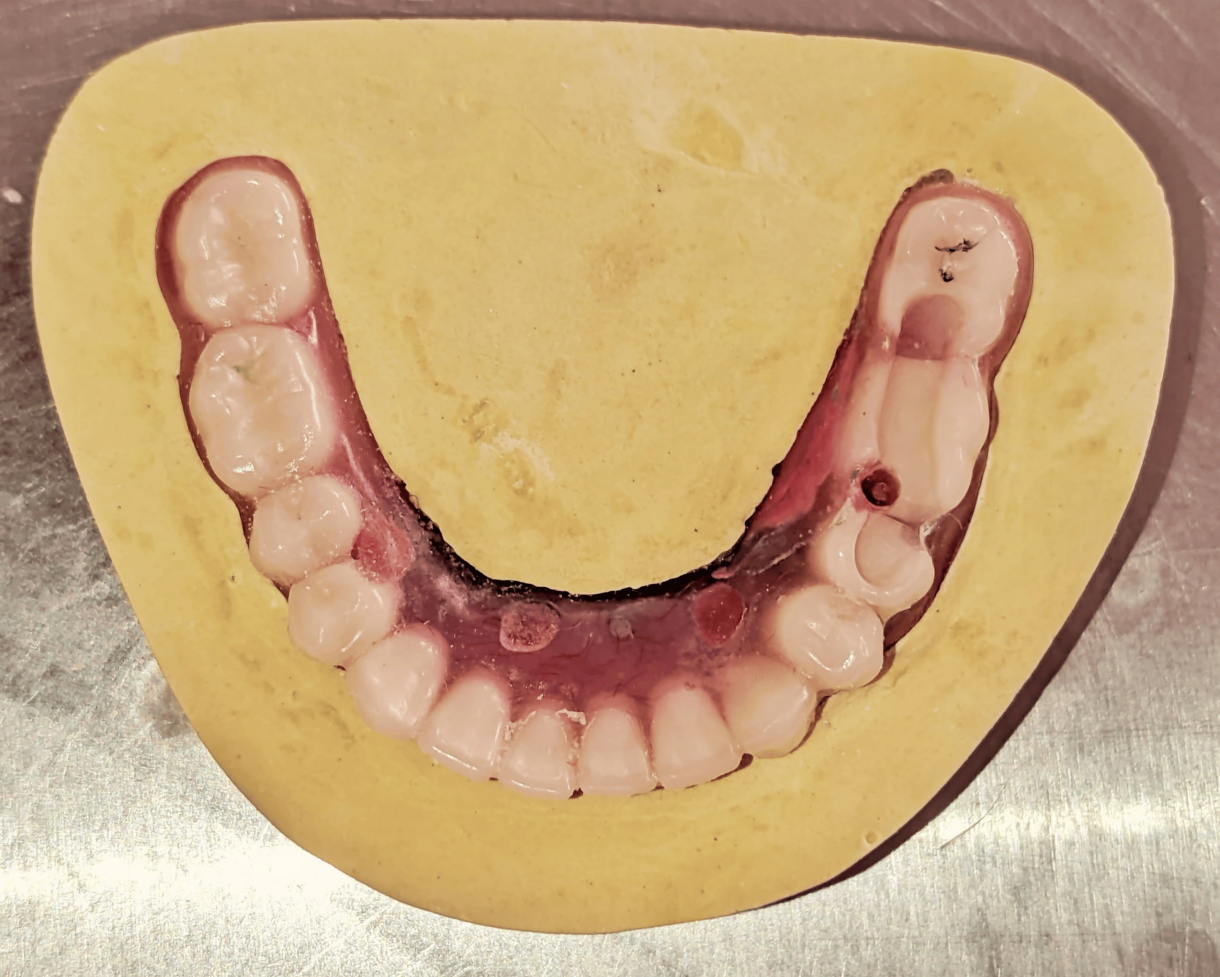
A tissue surface index is made, followed by inlay cavity preparation from the premolar to the mesial surface of the second molar.

Once the acrylic material was set, the prosthesis was separated from the occlusal index by sectioning the occlusal index. A tissue surface index (Figure 5) was made with the help of type III dental stone (gold stone type III).

**Figure 6:**
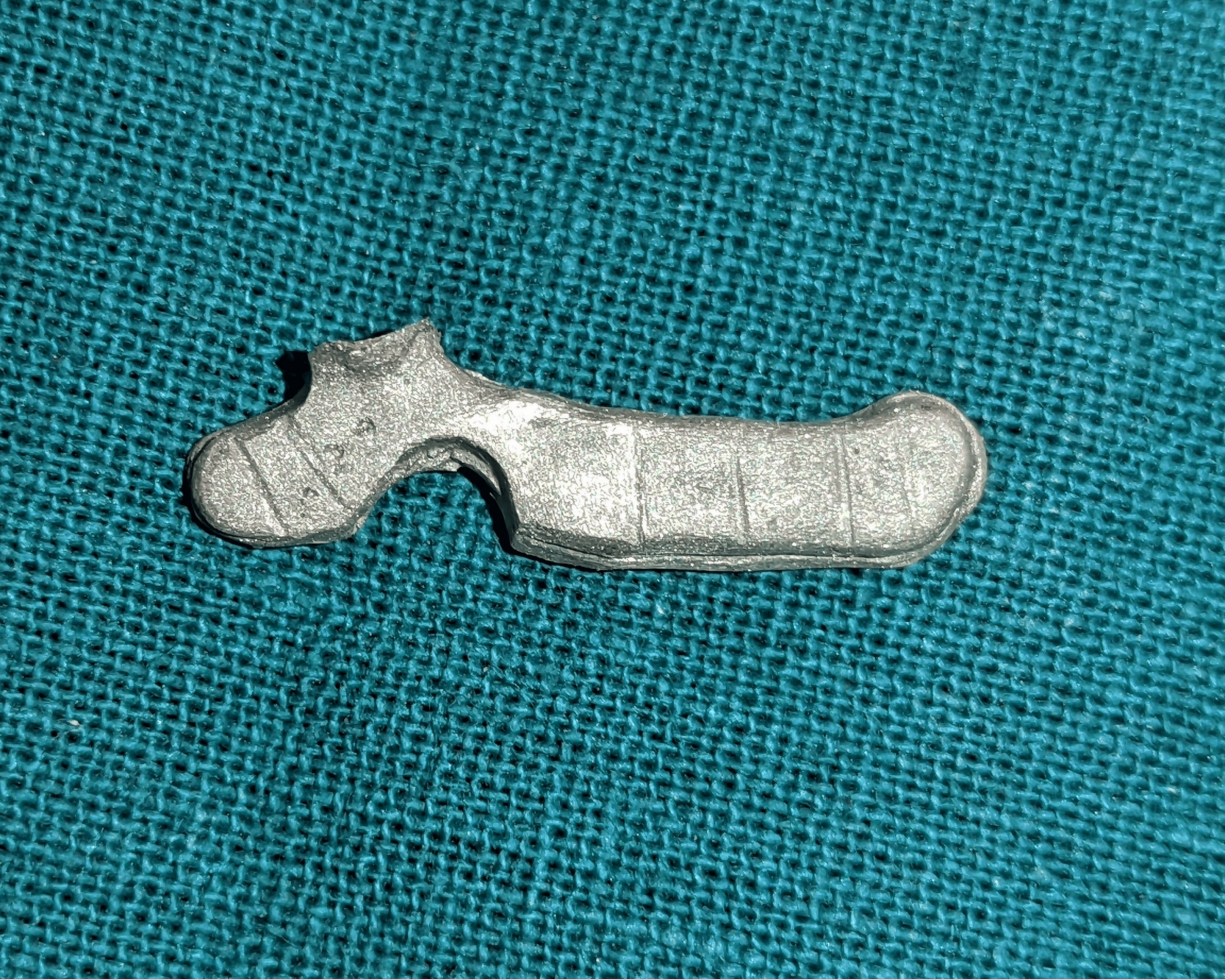
Metal inlay obtained from the wax pattern.

Putty (Elite HD+ Putty Soft) and light body (Elite HD+ Light Body) impression was made, and casts were poured with type III dental stone (gold stone type III). A layer of die harder, die spacer, and die lubricant was applied in the area of interest, and the wax pattern (2 GM Dental Inlay Wax; Giriraj Products, India) was fabricated for the inlay preparation. The wax pattern was retrieved and invested (GC FUJIVEST II; GC America, Alsip, IL). After the investment material solidified, it was put in a burnout furnace and then cast using an LC CAST 600T induction casting equipment (VOP Ltd., Botevgrad, Bulgaria). The casting was then retrieved, trimmed, and polished (Figure 6).

**Figure 7:**
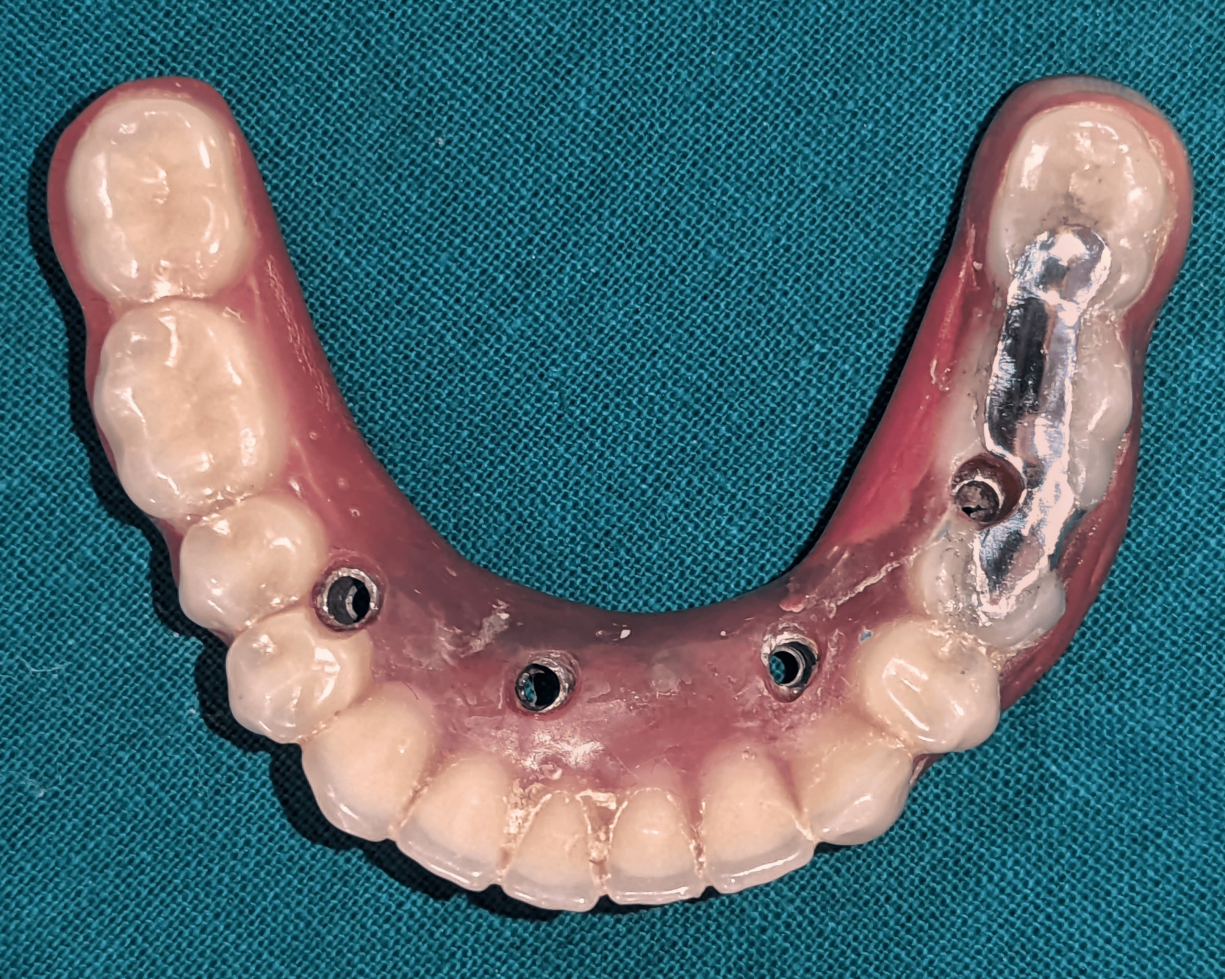
Metal inlay cemented with the help of cold cure acrylic.

The try-in of the prosthesis along with metal inlay was done, followed by cementing the inlay using cold-cure acrylic (Dental DPI RR Cold Cure), followed by finishing and polishing of the prosthesis (Figure 7).

**Figure 8:**
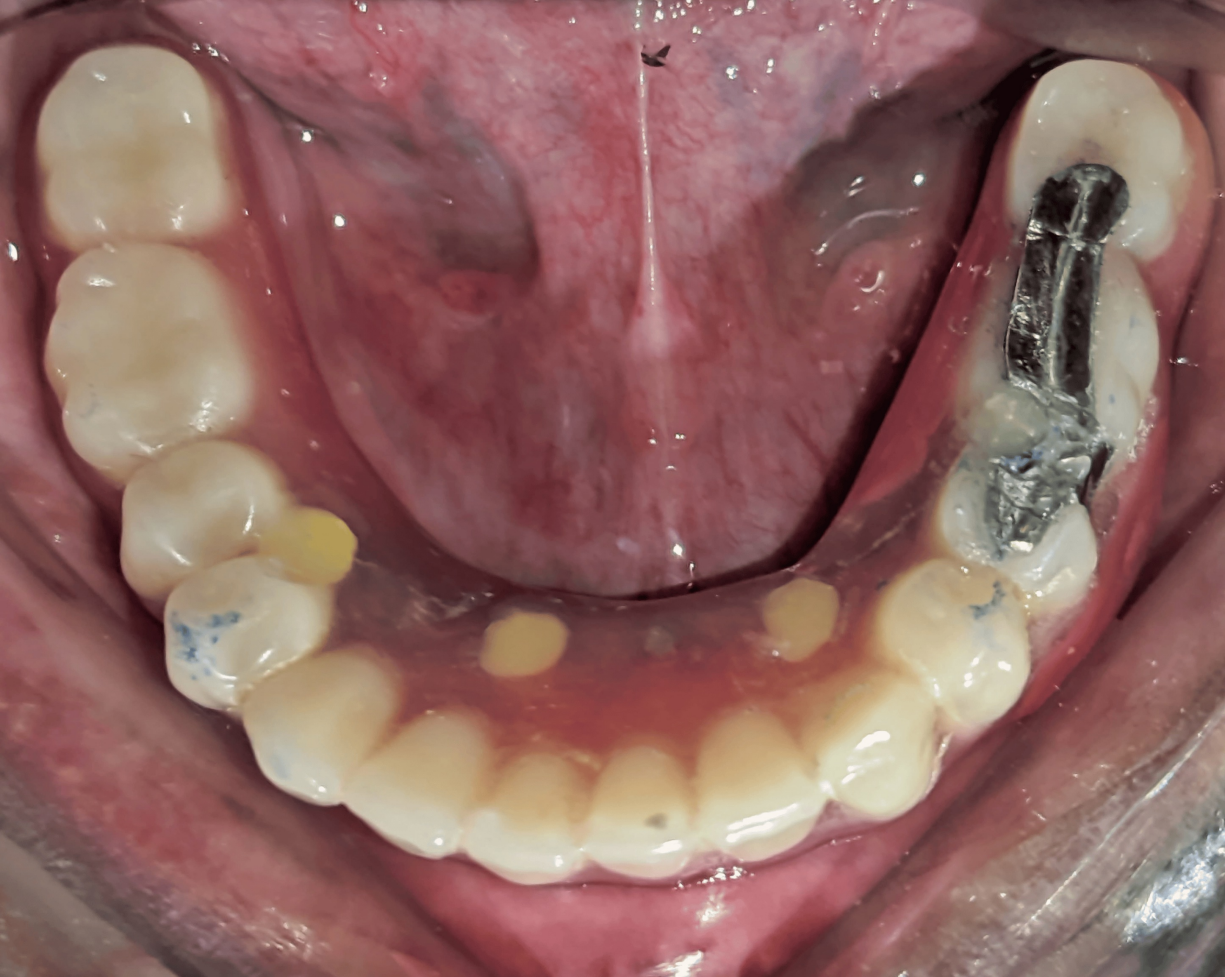
Final insertion of the corrected prosthesis intraoral.

**Figure 9:**
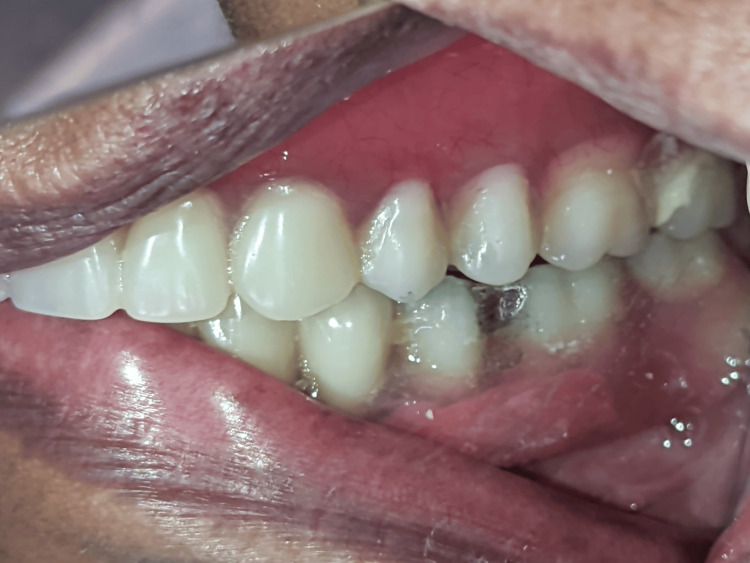
Occlusion was verified following the insertion of the prosthesis.

Once the fit of the mandibular denture was verified, the insertion of the mandibular prosthesis was done (Figure 8). Occlusion of the prosthesis was verified (Figure 9). There were no corrections made with respect to maxillary denture.

## Discussion

A common condition that affects millions of people worldwide is complete edentulism, or the loss of all teeth in one or both arches. Detachable implant-supported dentures and fixed implant-supported restorations are two possible treatment options. The patient's physical constraints and personal preferences will determine this course of treatment, which may involve undergoing significant surgery to replace the soft tissue and/or bone [4,5].

The term "osseointegration" describes the assimilation of an inorganic (metal) component into living bone. Osseointegration of endosseous dental implants was originally defined as a firm, direct, and long-lasting connection between vital bone and screw-shaped titanium implants with certain defined finishes and geometries [6,7].

Professionals are increasingly using implant-supported prostheses to perform oral rehabilitation procedures. Any prosthesis that lacks a traditional design and is typically made of several materials is referred to as a hybrid prosthesis [8].

A hybrid prosthesis often refers to fixed rehabilitation composed of a metal-based substructure covered with acrylic resin [9]. For edentulous patients who were unable to adjust to the long-term usage of conventional complete dentures, mandibular implant-supported hybrid prostheses have been utilized [10].

Although it is preferred to install as many implants as possible, hybrid prostheses can be created on a variable number of implants, with a minimum of four. This kind of prosthesis is thought to have improved edentulous patients' quality of life when compared to traditional full dentures since they provide psychological, functional, and cosmetic benefits [8].

The most common problems encountered with hybrid prostheses are wear of the denture teeth, fractures of the veneering acrylic or the denture teeth, lost fillings in screw-access openings, and mobile prostheses that are primarily caused by loose or broken screws.

Denture bases made of acrylic resin are preferred because they chemically bond to denture teeth and are easier to adjust [11]. Because of its many benefits, including biocompatibility, durability in oral conditions, pleasing aesthetics, low water sorption and solubility, affordability, and ease of processing and repair, acrylic resin made of poly (methyl methacrylate) (PMMA) is an organic biological material that is frequently used in the production of dental prostheses [12].

It is a frequent clinical occurrence for acrylic resin dentures to fracture [13]. In recent years, there has been a lot of focus on efforts to examine and identify the reasons for these fractures.

Denture repairs involve joining two parts of a fractured denture with a denture repair material. Repair of the denture base is accomplished by an auto-polymerized repair acrylic resin and grinding the surface of the denture base. Attempts to improve the bond strengths of denture base to repair acrylic resin have involved mechanical and chemical treatments [13].In a study conducted by Real-Osuna et al., they reported that the most common causes of failure in hybrid prosthesis were mucositis, followed by fractures of acrylic teeth [8]. Koshy et al., in their case report, have used malo bridge to repair a fractured hybrid denture [14].

The literature available with respect to the fracture of conventional dentures and methods to deal with it happens to be sufficient, but, in terms of hybrid dentures, not much information is available. In this case report, a simple, cost-effective way to repair the fractured portion of the acrylic denture of the hybrid prosthesis has been depicted.

The implementation of this method in clinical settings proves to be time- and cost-efficient, with the added benefit of reducing the frequency of patient appointments.

The limitation of this method can be that the treatment was limited to short-term management. This could be attributed to the underlying issue of not extending the prosthetic framework beyond the distal-most implant, which may have compromised load distribution and long-term success.

Cold-cure acrylic resin was used for the prosthesis, which is known to have potential drawbacks such as residual monomer release that may irritate the oral mucosa, especially in long-term use.

Aesthetic concerns may arise due to visible metal components in the prosthesis, which may affect patient satisfaction, particularly in cases involving high smile lines or anterior regions.

## Conclusions

Hybrid prostheses offer a valuable solution for patients who face difficulties adapting to conventional dentures, combining the stability of implants with the aesthetics and function of fixed restorations. However, despite their many advantages, complications such as fractures in the acrylic portion can compromise their longevity and patient satisfaction. Addressing this common issue with a straightforward, cost-effective repair method not only restores the prosthesis but also reinforces patient confidence in their treatment. By utilizing accessible materials and simple clinical techniques, dental professionals can minimize downtime and reduce the financial burden on patients. Ultimately, proactive maintenance and efficient repair strategies ensure that hybrid prostheses continue to deliver reliable, long-term outcomes in implant-supported restorative care. This case report depicts an effective way to restore a fractured hybrid prosthesis.
